# A Case Report Illustrating the Combined Use of Cryoneurolysis and Percutaneous Needle Tenotomy in the Treatment of Longstanding Spastic Shoulder Contractures After Stroke

**DOI:** 10.1016/j.arrct.2023.100285

**Published:** 2023-07-29

**Authors:** Samuel Herzog, Romain David, Abby Speirs, Mahdis Hashemi, Paul Winston

**Affiliations:** a Faculty of Science University of British Columbia, Vancouver BC, Canada; b Vancouver Island Health Authority, Victoria, BC, Canada; c Physical and Rehabilitation Medicine Unit, Poitiers University Hospital, University of Poitiers, 86021 Poitiers, France; d PRISMATICS Lab (Predictive Research in Spine/Neuromodulation Management and Thoracic Innovation/Cardiac Surgery), Poitiers University Hospital, Poitiers, France; e Division of Physical Medicine and Rehabilitation, University of British Columbia, Faculty of Medicine, Victoria, BC, Canada; f Canadian Advances in Neuro-Orthopedics for Spasticity Consortium, Kingston, ON, Canada

**Keywords:** Case report, Contracture, Denervation, Muscle spasticity, Rehabilitation, Tenotomy

## Abstract

Adduction and internal rotation of the shoulder is a common presentation in post-stroke patients, and can often be caused by spasticity and musculotendinous retraction causing a contracture of the pectoralis major and minor muscles. A post cerebral arteriovenous malfunction rupture patient with severe refractory left shoulder spasticity with contracture was treated with cryoneurolysis to the medial and lateral pectoral nerves, combined with a percutaneous needle tenotomy to the pectoralis major tendon. There was an improvement in shoulder forward flexion, abduction and external rotation immediately and found sustained at 8 weeks by 50°, 45°, and 15°. The patient noted an immediate cessation of limitation of shoulder abduction, a liberation of range of motion of the shoulder, and looseness in their arm and shoulder. They reported a dramatic improvement in their gait, increased independence, and an improvement in overall quality of life in a structured interview 8 weeks after the procedure. The patient relayed a positive experience with the combined neuro-orthopedic procedure of cryoneurolysis and tenotomy for the treatment of their spastic shoulder. This combined treatment could be considered as a management strategy for patients experiencing shoulder spasticity with contracture.

Upper limb spasticity is a common and disabling condition in patients after stroke, characterized as a velocity-dependent increase in muscle tone of the upper limb. 43% of stroke patients experience upper limb spasticity in the 12 months after diagnosis.^1^ A spastic shoulder is a common cause of hemiplegic shoulder pain in post-stroke patients and introduces a high risk of contracture.^2^ Spasticity and contracture of the shoulder can lead to decreased quality of life through significant impairments to shoulder function. Spastic shoulders with contracture require complex treatment plans, conventionally involving a surgical approach. Because of the neuro-orthopedic nature of the condition, separate interventions are necessary to treat both the spasticity and the contracture.^3^

Percutaneous cryoneurolysis is a novel, minimally-invasive treatment involving the ultrasound-guided application of a low temperature cryoneurolysis probe (cryoprobe) to a targeted peripheral nerve. The cryoprobe generates an ice ball at temperatures ranging between -66 and -88 °C out of interstitial fluids. The formation of the ice ball causes a limited zone of axon and myelin disruption, leaving the epineurium and perineurium intact and allowing the nerve a pathway to regenerate.^4^ Recent case studies show that cryoneurolysis continues to be effective for several months to years.^5^^,^^6^

Percutaneous needle tenotomy is a mini-invasive surgical technique in which a tendon sectioning is performed under short-acting anesthesia to treat contracture.^7^ Tenotomy is a well-established technique, originating in the 18th century, and has a lower risk of complications compared with more invasive open tendon surgeries.^8^

The use of cryoneurolysis and tenotomy in a single, combined procedure will be examined as a novel approach for the treatment of spastic shoulder.

## Case report

This study follows the Case Reports (CARE) guidelines and reports the required information accordingly (Appendix available online only at http://www.archives-pmr.org/). Institutional research ethics board approval was not required.

A 64-year-old women presents with left spastic hemiplegia secondary to a right sub frontal arteriovenous malfunction rupture 16 years prior. She has increased muscle tone in the left arm and leg, with left shoulder subluxation and muscle hypertonicity in the pectoralis major. These factors caused the patient's left arm to cross the midline of her body while walking, which caused her discomfort and interfered with her gait. The patient has a 10-year history of receiving Botulinum Toxin type A (BoNT-A) injections to the pectoralis major (40 units). She used an upper extremity sling brace to address the abnormal movement of her left arm, which was discontinued as it was ineffective. She uses an ankle-foot orthosis as well as a quad cane to ambulate. She also received BoNT-A injections to the left flexor digitorum longus (40 units) and gastrocnemius muscle (70 units). Prior to the cryoneurolysis and tenotomy procedures, she had long plateaued, making no further gains in achieving her goals for several years.

## Methods

The patient provided informed consent for this case report.

### Clinical evaluation

**Fig 1:**
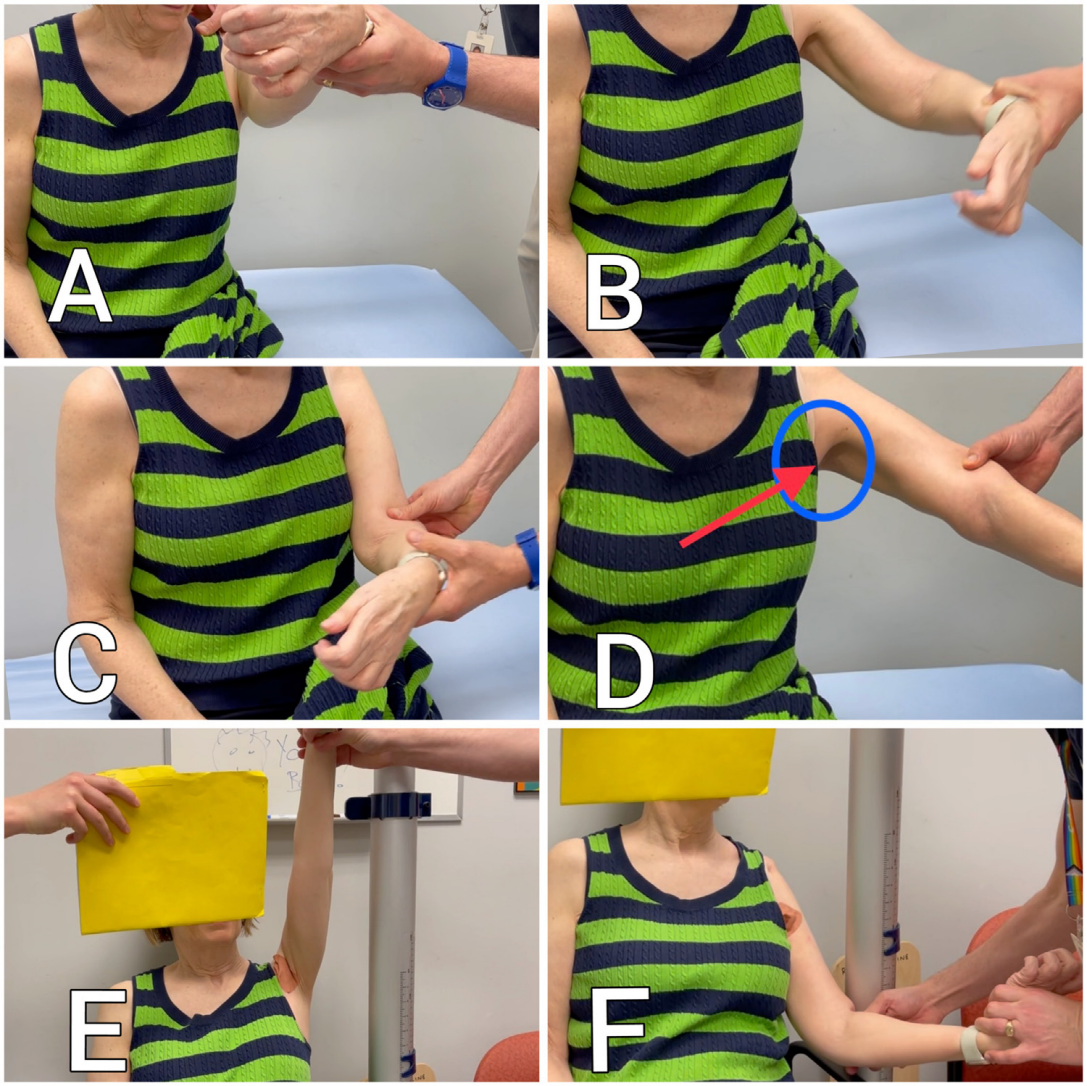
Baseline and post-procedural passive range of motion of the left shoulder. (A) Baseline shoulder forward flexion. (B) Baseline shoulder abduction. (C) Baseline shoulder external rotation. (D) Abduction with straight elbow exposes pectoral tendon (arrow). (E) Post procedure shoulder abduction and flexion have the same endpoint. (F) Post procedure shoulder external rotation.

On clinical evaluation, it was determined that the pectoralis major and minor muscle were most likely implicated in the patient's spastic shoulder adduction and internal rotation (fig 1A-C). A diagnostic nerve block was not performed because of the awareness that there was a spastic component that responded to BoNT-A as well as a palpable musculotendinous retraction with the presence of an evident contracture of the pectoralis major muscle (fig 1D). Other muscles commonly involved in the spastic shoulder deformity were determined not to be implicated by the examining physician, via palpation of the latissimus dorsi and subscapularis in the axilla while the arm was ranged. It was decided that the medial and lateral pectoral nerves would be the first target for cryoneurolysis, with further muscles or tenotomy of the pectoralis major to also immediately be performed if the initial cryoneurolysis was not sufficient for the patient's goals.

### Interventional procedure

The patient was warned of potential side effects and complications, and consented to cryoneurolysis of the medial and lateral pectoral nerves. She also underwent cryoneurolysis to the intramuscular branches of the medial and lateral gastrocnemius, and flexor digitorum longus for mild spasticity.

Baseline measurements of the left shoulder were taken of active and passive range of motion (ROM) on the Modified Tardieu Scale, and spasticity was assessed on the Modified Ashworth Scale (MAS) (table 1). The patient was prepared with 4% chlorohexidine to the skin, and the skin at the site of the injection was anesthetized with 1% lidocaine. Then, a 16-gauge angiocatheter was inserted into the skin to prevent frostbite and facilitate insertion. The nerves were targeted with ultrasound and e-stimulation guidance. The hand-held cryoprobe^a^ was inserted through the catheter, and the probe was placed in close proximity with each targeted nerve. Two lesions were formed for the medial and lateral pectoralis nerves.

**Table 1:** Shoulder parameters before and after treatment

Baseline	V1	AROM	MAS*
Left shoulder flexion (degrees)	95	None	2
Left shoulder abduction (degrees)	90	None	2
Left shoulder external rotation (degrees)	35	None	1+
8 weeks after cryoneurolysis/tenotomy	V1	AROM	MAS
Left shoulder flexion (degrees)	145	None	1+
Left shoulder abduction (degrees)	135	None	1+
Left shoulder external rotation (degrees)	50	None	1

Abbreviation: AROM, Active Range Of Motion.

⁎ **Measured within the patient's available range of motion.**

**Fig 2:**
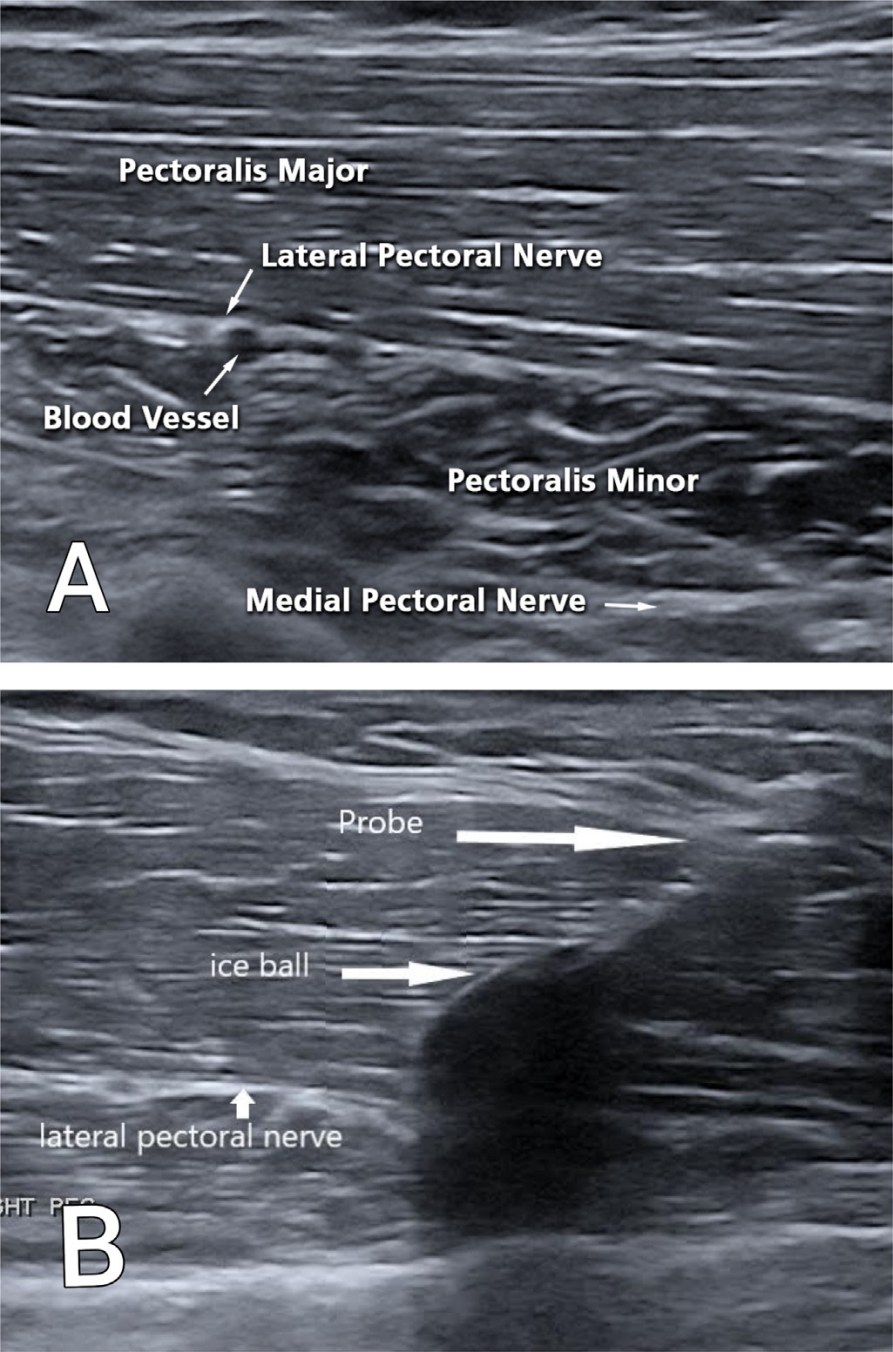
(A) Anatomy of the lateral and medial pectoralis nerves. (B) Cryoneurolysis of the lateral pectoral nerve.

Cryoneurolysis of the left medial and lateral pectoral nerves was performed (fig 2). Directly after the procedure, the patient experienced an immediate increase of 20° in ROM in the shoulder with forward flexion, abduction and improved external rotation with a drop in tone on the MAS. When her shoulder was passively flexed or abducted to its new maximum range, the patient experienced pain, and the examining physician noted even more prominence of the pectoralis major tendon. The patient then consented to the tenotomy procedure in the event that further intervention was deemed necessary. For the tenotomy procedure, an ultrasound localization was performed to optimize the site of treatment at the distal part of the pectoralis major tendon. This is found at the anterior fold of the axilla as a palpable and visual tendon (fig 1D). Next, the target area was anesthetized with 3 cc of 1% lidocaine, and the lower part tendon to the pectoralis major, responsible for adduction and internal rotation, was fenestrated with an 18-gauge needle. The tenotomy was performed to the lower part of pectoralis major responsible for adduction and internal rotation of the shoulder.

The patient was satisfied with the result, and the other muscles did not require treatment.

## Results

Immediately after the combined procedure, the patient's ROM in her shoulder increased significantly (fig 1 and video). She also reported a sense of looseness, and a cessation of pain in her pectoralis region. She noted light-headedness and nausea after the procedure but recovered quickly. She otherwise tolerated the procedure very well. At a follow-up appointment 8 weeks after the procedure, the patient reported a sustained cessation of the shoulder adduction and internal rotation, as well as looseness in their arm and shoulder, and an improvement in her gait. She also described a cessation of a previous pulling pain in her pectoralis minor tendon. Measurements of the patients shoulder 8 weeks after the procedure were collected (table 1). An interview with the patient was conducted 8 weeks after the procedure (table 2).

**Table 2:** Summary of the interview with the patient 56 days after treatment

How does your experience with botulinum toxin injections compare with your experience with cryoneurolysis?	“The toxin injection is less painful but the effects wore off fairly quickly. This [cryoneurolysis] seems to be lasting way longer.”
Did you find that the cryoneurolysis procedure was painful?	“The cryoneurolysis was more painful but not terrible. I did, however, feel like I was going to throw up when they did the cryoneurolysis in the shoulder.”
Have you noticed any side effects from the procedure?	“I had no side effects. There didn't seem to be anything at all. No bruising or swelling.”
Did you notice any changes immediately after the cryoneurolysis?	“Yes. Right away I could immediately see and feel that my arm was a lot looser.”
How are you managing with your activities of daily living?	“It is now easier to get dressed and easier to wash under my arm. [Since the procedure] I have no pain whereas I used to have pain especially when I got tired.
Prior to treatment, you were experiencing difficulty with your gait because your elbow was flexed and your arm was internally rotated. How is that now?	“Tremendous. The arm just hangs down now which is so much better. Before when it started to pull up it would affect my balance.”
Is there anything that you couldn't do before the procedure that you can do now?	“Yes. Now I can go all over the playgrounds and the parks with my grandkids. I can walk several kilometers, I do get tired, but I'm not in pain anymore.”
This is a novel procedure, do you have any suggestions on how to improve the experience?	“The use of the freezing spray was helpful for me and it should definitely be a part of the procedure going forward. I also think that it would be nice to have something like ginger ale on hand for when people feel nauseous.”
Are you happy with your results from the procedure?	“I am delighted. It's an improvement in quality of life which is critical for people like me. Do you know how liberating it is to be able to shave your underarm? It's a big step for me.”
Did you notice any changes immediately after the tenotomy? Did you feel any release or increased freedom of movement?	“I did, yes. There wasn't the expected pull anymore. I could feel right away that it was looser. They repeated all of the measurements right away and it was obvious to me that there was a distinct improvement.”
How did the tenotomy help you?	“I used to have a lot of pulling pain right where that tendon is but now I don't have any of that, even after a long day, which is remarkable given that my arm is also swinging more now.”

## Discussion

A 64-year-old patient presented with a spastic deformities of the shoulder as well as muscle tone in the pectoralis major muscle. Her improvement had plateaued despite several years of conventional treatment with BoNT-A and physiotherapy. As a result of the cryoneurolysis procedure, there was a significant improvement on the MAS scale for her shoulder movements. All improvements were maintained at 8 weeks post-procedure. She also commented on her improved gait. She attributed this more the increased arm swing than the lower leg treatment for mild spasticity in the leg.

The pectoralis major is a large muscle of the anterior chest wall. It contributes to shoulder flexion, adduction, and internal rotation. It is composed of 3 heads: the clavicular, sternal, and abdominal head, and it is innervated by medial and pectoral nerves. The clavicular head of the pectoralis major is implicated mainly in the flexion of the shoulder and is innervated by the lateral pectoral nerve. The sternal head is mainly implicated in shoulder adduction and is innervated by the medial pectoral nerve. The abdominal head is implicated in shoulder internal rotation and is innervated by the medial pectoral nerve.^9^ Based on this knowledge, we treated the spasticity causing adduction and internal rotation of the shoulder by targeting the medial and lateral pectoral nerves for cryoneurolysis, as well as the contracture by treating the lower and distal segment of the pectoralis major tendon via tenotomy.

In cases of upper limb spasticity, it is often difficult to determine which possible factor is limiting joint movement. When spasticity causes contracture, a focal approach such as cryoneurolysis must be combined with an orthopedic procedure in order to restore ROM to the implicated limb.^2^^,^^3^ Several established surgical interventions exist to treat contracture, such as tendon or muscle lengthening. These procedures are invasive, and must be performed under general anesthesia. Because medical frailty is a barrier to surgical intervention for many post-stroke spasticity patients, these procedures are often not feasible. Tenotomy provides a well-studied, safe, and minimally-invasive alternative to open-tendon surgery. Both the tenotomy and cryoneurolysis procedures can be performed in any setting, providing a more accessible treatment plan to patients with impaired mobility.^4^^,^^8^ Additionally, because tenotomy and cryoneurolysis are minimally-invasive treatments which do not require the use of general anesthesia, they can be applied to treat medically frail upper limb spasticity patients with contractures, a population which often lacks effective treatments.^10^

### Limitations

As a case study with a sample size of 1, this study is not fully representative of the diverse experiences of post-stroke patients. Additionally, as both cryoneurolysis and tenotomy were performed on the same day, only a qualitative comparison could be made between the patients’ full ROM after the cryoneurolysis procedure and her ROM after both cryoneurolysis and tenotomy.

## Conclusions

Cryoneurolysis of the left medial and lateral pectoral nerve and tenotomy of the pectoralis major tendon were performed on a 64-year-old patient who reported sustained improved passive ROM, reduced pain, and increased mobility during a structured interview at an 8-week follow-up. These improvements suggest that this minimally-invasive, single treatment combined procedure was effective in addressing spasticity and contracture in this patient. Further studies are necessary to verify the effectiveness of a neuro-orthopedic treatment plan and should explore possible applications to medically frail patients.

## Supplier

Iovera System 190 Smart Tip; Iovera, Pacira

## Appendix Supplementary materials

Image, video 1
